# Apathy in Neuropsychiatric Disorders: Clinical Characteristics, Neurobiological Mechanisms, and Therapeutic Strategies

**DOI:** 10.1155/bn/3788122

**Published:** 2025-09-15

**Authors:** Ozlem Totuk, Sevki Şahin

**Affiliations:** Department of Neurology, Hamidiye Faculty of Medicine, University of Health Sciences, Sancaktepe Sehit Prof. Dr. Ilhan Varank SUAM, Istanbul, Türkiye

**Keywords:** apathy, cognitive and behavioral symptoms, motivational deficits, neurobiology of apathy, neuropsychiatric disorders

## Abstract

Apathy is a prevalent yet frequently underrecognized neuropsychiatric syndrome characterized by diminished motivation and reduced goal-directed behavior across multiple domains. It is strongly associated with poorer functional outcomes, increased caregiver burden, and decreased quality of life in various neuropsychiatric conditions. Despite its clinical importance, apathy remains underdiagnosed and undertreated, partly due to overlapping features with depression and cognitive impairment. This narrative review synthesizes current knowledge on conceptualization, neurobiological mechanisms, diagnostic criteria, and management strategies for apathy, adopting a transdiagnostic perspective across disorders such as Alzheimer's disease, Parkinson's disease, frontotemporal dementia, multiple sclerosis, and major psychiatric conditions. This review distinguishes itself by integrating subtype-based approaches, biomarker insights, and emerging digital tools, providing a framework for more precise characterization and personalized intervention. This review is based on a nonsystematic literature search conducted in PubMed, Scopus, and Google Scholar for articles published between 2011 and 2025. Improved characterization and management of apathy are essential for optimizing patient outcomes, reducing caregiver burden, and guiding future research.

## 1. Introduction

Apathy is a prevalent and clinically significant behavioral syndrome characterized by a persistent reduction in motivation, which is observed across a broad spectrum of neurological and psychiatric disorders [1]. Its clinical presentation typically includes diminished goal-directed behavior, reduced emotional responsiveness, and a lack of initiation in daily activities [1–4]. One of the defining features of apathy is the loss of self-initiated action, which differentiates it from related conditions such as depression and fatigue [5–7]. For diagnostic clarity, symptoms must persist for at least 4 weeks and represent a notable decline from the individual's prior level of functioning [7].

Despite its high prevalence, apathy is often underrecognized and misattributed to other syndromes, especially depression. Unlike depression, which is marked by dysphoria, guilt, and hopelessness, apathy is defined by emotional neutrality and diminished motivation in the absence of sadness [3]. It is also frequently confused with fatigue and cognitive impairment, yet it remains a distinct syndrome with its own neurobiological and clinical profile.

The impact of apathy is substantial. It contributes to reduced quality of life, increased caregiver burden, and worsened functional outcomes in conditions such as Alzheimer's disease (AD), Parkinson's disease (PD), frontotemporal dementia, stroke, schizophrenia, and multiple sclerosis (MS) [6, 8, 9]. It also has prognostic significance, being associated with accelerated cognitive and functional decline and reduced rehabilitation potential [10, 11]. Social isolation, such as that experienced during the COVID-19 pandemic, may further exacerbate apathetic symptoms [12].

The underdiagnosis of apathy stems partly from the lack of standardized diagnostic tools and limited multidisciplinary awareness. Clinicians often rely on observational data, informant interviews, and clinical rating scales, yet there is no universally accepted diagnostic gold standard [3]. Additionally, the distinction between primary apathy and secondary motivational deficits caused by depression or cognitive fatigue is frequently blurred.

This article presents a narrative review synthesizing the clinical characteristics, neurobiological mechanisms, and therapeutic approaches to apathy across diverse neuropsychiatric disorders. The diagnosis of apathy primarily relies on clinical evaluation, including interviews with patients and caregivers, behavioral observation, and the use of validated scales. This approach is essential for distinguishing apathy from comorbid conditions such as depression and fatigue. Literature was selected through a nonsystematic search of peer-reviewed sources published between 2011 and 2025, using databases such as PubMed, Scopus, and Google Scholar. Keywords included apathy, neuropsychiatric disorders, motivation, and nonmotor symptoms.

What distinguishes this review from previous works (e.g., [8–10]) is its transdiagnostic approach, which highlights shared and divergent mechanisms of apathy across conditions. Furthermore, the review emphasizes subtype-specific features (behavioral, cognitive, and emotional) and incorporates emerging perspectives including digital phenotyping, biomarker-informed subtyping, and technological interventions. The goal is to offer an integrative framework that bridges clinical assessment with underlying neurobiology and points toward more personalized, multidimensional treatment strategies.

## 2. Subtypes of Apathy

Apathy is increasingly understood as a multidimensional construct comprising distinct yet interrelated domains. It is commonly categorized into three core subtypes—behavioral, cognitive, and emotional—each associated with dysfunction in specific neural circuits [9]. This typological framework enables clinicians and researchers to better characterize the underlying mechanisms of apathy in various neuropsychiatric conditions.

Behavioral apathy reflects reduced self-initiated actions despite preserved motor capacity and is linked to mesolimbic dopaminergic dysfunction (e.g., ventral tegmental area and nucleus accumbens).

Cognitive apathy involves motivational deficits in executive planning and decision-making, associated with the dorsolateral prefrontal cortex (DLPFC) and frontostriatal circuitry.

Emotional apathy is characterized by blunted affect and reduced empathy, often related to the orbitofrontal cortex (OFC), anterior cingulate cortex (ACC), and ventral striatum (VS).

Although these subtypes frequently coexist, identifying the dominant apathy profile in each patient can inform more tailored clinical assessments and intervention strategies. This approach is particularly relevant because each subtype may correspond to distinct neurochemical and structural correlates, which in turn may predict response to pharmacological or behavioral therapies [13].

Table 1 provides a summary of the neuroanatomical correlates and primary clinical manifestations associated with each apathy subtype.

This multidimensional model of apathy has significant implications for diagnosis, treatment planning, and research design. Future studies should explore how subtype-specific presentations interact with disease pathophysiology, treatment response, and patient outcomes.

## 3. Neurobiological Foundations of Apathy

Apathy is increasingly recognized as a biologically grounded syndrome, arising from dysfunction in distributed brain circuits rather than a purely psychological phenomenon [10–12]. Neuroimaging, neuropathological, and neurochemical evidence converge on the involvement of frontal–subcortical systems, large-scale brain networks, and key neurotransmitter pathways.

### 3.1. Frontal–Subcortical Circuits

**Figure 1:**
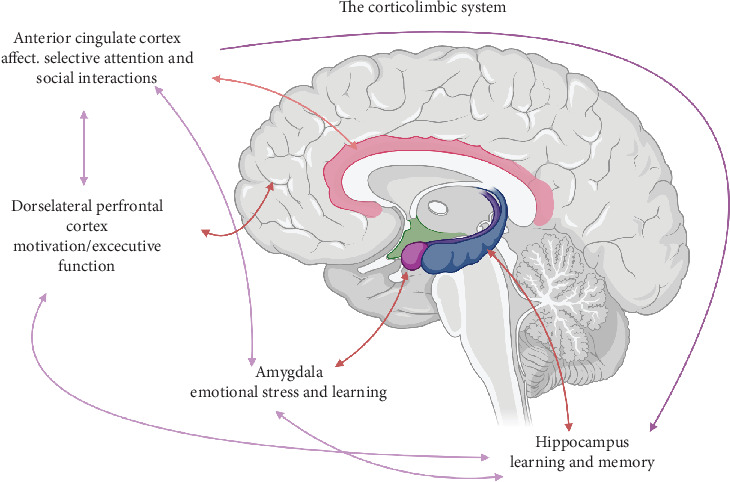
Schematic representation of frontal–subcortical circuits.

Frontal–subcortical loops, particularly those linking the prefrontal cortex and basal ganglia, play a critical role in motivation and goal-directed behavior [14]. Three key circuits—sensorimotor, associative, and limbic—connect regions such as the DLPFC, OFC, and ACC to the striatum via dopaminergic pathways [14, 15]. Dysfunction within these networks produces core apathetic features: amotivation, cognitive inertia, and autoactivation deficits [15]. Neuroimaging supports these associations, revealing structural and metabolic changes in these regions among apathetic patients. These pathways and their interconnections are illustrated schematically in Figure 1.

### 3.2. Large-Scale Brain Networks

Beyond localized lesions, apathy is linked to disruptions across large-scale networks, including the cognitive control network (DLPFC, dorsal ACC, and dorsal caudate), salience network (OFC, vmPFC, and anterior insula), and default mode network (mPFC, PCC, and hippocampus). These systems govern executive control, salience detection, and self-referential processing [10]. Altered connectivity within these networks, particularly involving the vmPFC, contributes to the multidimensional nature of apathy [16].

### 3.3. Integrated Model and Clinical Relevance

Apathy emerges from combined dysfunction in corticostriatal loops, PFC–basal ganglia interactions, and disrupted network connectivity [6, 10]. This integrated view links behavioral, cognitive, and emotional subtypes to overlapping but distinct neural substrates, informing a multidimensional diagnostic and therapeutic framework. Major brain regions implicated in apathy are summarized in Table 2.

### 3.4. Neurochemical Systems

Multiple neurotransmitter systems contribute to the pathophysiology of apathy:

• *Dopaminergic system*: Central to reward processing and effort-based decision-making; deficits, especially in D3 receptor activity, are strongly implicated in apathy in PD and AD [17–20].
• *Noradrenergic system*: Degeneration of the locus coeruleus correlates with apathy in neurodegenerative conditions [21–24].
• *Cholinergic system*: Dysfunction in the nucleus basalis of Meynert contributes to attentional deficits and cognitive apathy [15].
• *Serotonergic system*: May influence emotional responsiveness via ACC pathways, though findings remain inconsistent [6].

These systems interact in complex ways; for instance, noradrenergic tone modulates cholinergic activity, and dopaminergic depletion alters cortical activation thresholds.

## 4. Differential Diagnosis and Assessment Scales

### 4.1. Differential Diagnosis and Conceptual Clarifications

The diagnosis of apathy is essentially clinical, relying on detailed anamnesis, interviews with the patient and caregivers, behavioral observation, and standardized assessment tools. Apathy is frequently misinterpreted as depression, fatigue, or cognitive impairment due to overlapping features. However, it is a distinct syndrome with unique phenomenological and neurobiological characteristics [3, 6].

Depression, though often comorbid, is defined by persistent sadness, guilt, and hopelessness—features absent in apathy. In contrast, apathetic individuals exhibit emotional indifference rather than emotional distress, a distinction captured by the phrase: “not sad, just indifferent.”

Fatigue denotes a subjective feeling of exhaustion, while motivational drive is generally preserved. Similarly, cognitive impairment and dementia can mimic apathy but remain conceptually separate; apathy may independently contribute to functional decline.

Distinguishing apathy from these conditions is essential for accurate diagnosis and treatment planning. Misclassification can lead to inappropriate interventions and delayed care.

Key distinguishing features are listed in Table 3.

### 4.2. Diagnostic Criteria

The diagnostic criteria for apathy (DCA) in neurocognitive disorders, published by Miller et al., provide a standardized framework for diagnosing apathy [3]. The criteria include the following:

• A significant and sustained reduction in goal-directed behavior or cognitive activity for at least 4 weeks
• Impairment in at least two of the following domains: behavioral/cognitive functioning, emotional engagement, and social interaction
• A noticeable functional impact and decline from the individual's prior level of functioning
• Symptoms that cannot be better explained by other medical, psychiatric, or neurological conditions

These criteria offer a valuable basis for improving diagnostic accuracy, particularly in dementia and other neurodegenerative disorders.

### 4.3. Clinical Assessment Tools

A range of scales has been developed to evaluate apathy, varying in dimensional coverage, validation status, and clinical utility [25, 26].

The Apathy Evaluation Scale (AES), developed by Marin, is among the most widely used tools and captures behavioral, cognitive, and emotional domains [2].

The Lille Apathy Rating Scale (LARS) offers structured interviews and demonstrates strong psychometric validity, particularly in PD and frontotemporal dementia [27].

The Neuropsychiatric Inventory (NPI) includes an apathy subscale but provides only a unidimensional assessment, limiting its specificity [28].

The Dimensional Apathy Scale (DAS) was developed to differentiate cognitive, behavioral, and emotional apathy, making it especially valuable in research and clinical practice [29].

The Apathy Motivation Index (AMI) is a recently proposed self-report tool that aligns with the multidimensional nature of apathy and is under active validation [30].

Combining caregiver and clinician input remains critical, especially in populations with cognitive impairment or limited insight. Discrepancies across informants should prompt behavioral observation and multi-instrument assessment.

Commonly used apathy scales are presented in Table 4.

## 5. Apathy in Neuropsychiatric Disorders

Apathy is a prevalent and clinically significant symptom across a range of neuropsychiatric disorders. Its presentation varies by etiology but typically predicts worse functional outcomes, caregiver burden, and accelerated disease progression. Tables 5 and 6 summarize prevalence, core clinical features, and neurobiological correlates across disorders.

### 5.1. Frontotemporal Spectrum Disorders (bvFTD and ALS)

Apathy is a core diagnostic feature of bvFTD and one of the earliest symptoms, often preceding disinhibition or stereotypies [31, 32]. Patients demonstrate severe loss of initiative and emotional engagement, frequently requiring caregiver input due to poor insight [33]. Its early emergence in bvFTD often leads to diagnostic confusion with depression; however, emotional indifference rather than dysphoria is the hallmark feature.

In ALS, apathy occurs independently of motor dysfunction and correlates with reduced quality of life and adherence to supportive interventions. Cognitive screening often reveals executive dysfunction coexisting with apathy, which may predict earlier ventilatory support needs. Neuroimaging reveals degeneration in the medial PFC, ACC, OFC, and striatal regions, which disrupt effort-based decision-making [34, 35].

### 5.2. Schizophrenia

Apathy is a prominent negative symptom in schizophrenia, present in over half of patients [36, 37]. Unlike in dementia, apathy in schizophrenia strongly affects social functioning and treatment adherence. Persistent apathy has been associated with poorer community integration and lower likelihood of competitive employment, even after positive symptom remission. Neurobiological findings implicate structural and functional disruptions in the DLPFC, ACC, and basal ganglia, aligning with deficits in salience and cognitive control networks [14].

### 5.3. Major Depressive Disorder (MDD)

Although often conflated with anhedonia, apathy in MDD represents a distinct motivational deficit [3, 6]. Patients frequently report: “I'm not sad, I just don't feel like doing anything.” This dissociation has prognostic implications, as individuals with predominant apathy tend to exhibit poorer antidepressant response and require adjunctive interventions. Neuroimaging shows hypometabolism in the VS, ACC, and mPFC; arterial spin labeling (ASL) studies associate reduced dorsal ACC perfusion with apathy severity [14, 15].

### 5.4. MS

Apathy affects 20%–50% of individuals with MS [38] and is often underrecognized. It correlates with executive dysfunction and demyelination in frontal–subcortical pathways, particularly DLPFC and basal ganglia [14]. Longitudinal data suggest that apathy predicts faster cognitive decline and greater disability accumulation, making early recognition clinically critical. Pharmacological options remain limited; structured exercise and cognitive stimulation show preliminary benefits [39, 40].

### 5.5. PD

Apathy in PD affects 20%–60% of patients [41, 42] and predicts faster cognitive decline [26, 43]. It stems from mesolimbic dopaminergic dysfunction involving the VTA and VS [6, 41], distinct from nigrostriatal motor pathways. Apathy often persists despite optimal motor symptom control and may worsen during deep brain stimulation, underscoring its complex pathophysiology. Clinical impact includes poor medication adherence and caregiver distress [43].

### 5.6. Huntington's Disease (HD)

Present in up to 70% of HD patients, apathy often emerges in the prodromal stage [44]. It reflects early striatal and thalamocortical degeneration, disrupting motivational circuitry [7, 44]. Severe apathy has been linked to reduced participation in cognitive rehabilitation, further accelerating functional loss. Poor patient insight necessitates clinician and caregiver assessment [45, 46].

### 5.7. Stroke

Poststroke apathy affects 20%–60% of survivors and significantly impedes rehabilitation [47, 48]. Lesions in the medial frontal cortex, ACC, DLPFC, and basal ganglia are commonly implicated [49]. Its persistence into the chronic phase predicts reduced independence and poorer quality of life, even after motor recovery. Early detection is crucial to enhance engagement and recovery outcomes.

### 5.8. AD

Apathy is one of the most disabling behavioral symptoms in AD, with prevalence up to 80% [50]. It predicts transition from MCI to dementia and correlates with tau pathology and ACC/VS dysfunction [51–54]. Clinically, apathetic patients are more likely to exhibit rapid functional decline and require earlier institutional care. Treatment remains challenging: Cholinesterase inhibitors provide modest benefit, while methylphenidate and repetitive transcranial magnetic stimulation (rTMS) show promising adjunctive effects [39, 40, 55–58].

## 6. Treatment Approaches and Clinical Recommendations

Currently, there is no universally approved pharmacological treatment specifically indicated for apathy. Management remains largely symptomatic and condition-specific, requiring individualized, multidisciplinary strategies that combine pharmacological and nonpharmacological approaches.

### 6.1. Pharmacological Interventions

Although apathy is frequently measured as a secondary endpoint, few trials have targeted it as a primary outcome. Evidence supports the following pharmacological strategies:

• Dopamine agonists (e.g., ropinirole and pramipexole): Effective in PD; enhance reward sensitivity via D2/D3 receptor stimulation [59].
• Cholinesterase inhibitors (e.g., donepezil and rivastigmine): Provide modest benefit in AD and PD dementia, though functional gains are limited [55].
• Methylphenidate: Reduces apathy in AD in short-term trials; effects on quality of life remain inconsistent [56, 57].
• Modafinil: Improves wakefulness and attention in MS and AD; long-term efficacy for apathy is unproven [39].
• Bupropion: A norepinephrine–dopamine reuptake inhibitor with mixed results across MDD, MS, and AD [40].

Evidence from pharmacological interventions is summarized in Table 7.

### 6.2. Nonpharmacological Interventions

Nonpharmacological interventions remain the cornerstone of apathy management and should be prioritized as first-line strategies:

• Exercise programs: Structured physical activity, including aerobic and resistance training, improves motivation and daily functioning in PD and AD [26].
• Cognitive stimulation therapy (CST): Enhances executive function and engagement, particularly in AD [10].
• rTMS: Targeting prefrontal cortex, rTMS shows significant and durable improvements in apathy symptoms, with effects persisting for up to 4 years [58].
• Environmental modifications: External cueing, enriched settings, and routine structuring reduce passive behavior, especially in dementia care.
• Digital therapeutics and emerging technologies: Virtual reality (VR), mobile applications, and wearable sensors enable both assessment and intervention. VR-based cognitive engagement platforms demonstrate improvements in goal-directed behavior among individuals with mild cognitive impairment [60]. Wearable accelerometers provide objective activity metrics in PD, correlating with apathy severity [61]. These scalable, noninvasive tools support personalized and data-driven apathy management.

### 6.3. Clinical Recommendations for Practice

To ensure systematic and individualized care, the following principles are recommended:

• Early detection: Integrate validated tools (AES and DAS) into routine assessments.
• Accurate differentiation: Distinguish apathy from depression and fatigue to avoid misclassification and inappropriate treatment.
• Prioritize nonpharmacological interventions: Behavioral activation, structured exercise, and environmental strategies should precede pharmacological approaches in mild cases.
• Adopt a subtype-specific framework: Tailor treatment to the dominant apathy subtype—behavioral, cognitive, or emotional.
• Continuous monitoring: Track symptom progression and functional performance with structured tools to guide timely adjustments.

Structured recommendations are provided in Table 8.

## 7. Future Perspectives

Despite increasing recognition of its clinical significance, apathy remains underinvestigated as a primary therapeutic target. Most clinical trials still evaluate apathy as a secondary endpoint, limiting progress in developing tailored treatments and diagnostic tools. Bridging this gap requires a multidimensional research agenda integrating clinical neuroscience, digital health, and personalized care models.

### 7.1. Biomarker-Based Classification

Advances in neuroimaging and fluid biomarkers provide a foundation for more precise apathy subtyping, particularly in neurodegenerative disorders. Reduced perfusion in the ACC, detectable by ASL, correlates with motivational deficits [14, 15]. In AD, tau burden strongly predicts apathy severity, supporting biomarker-driven diagnostic stratification [51, 52].

Longitudinal studies combining amyloid/tau PET imaging and cerebrospinal fluid profiling could identify patients at risk for severe apathy and guide subtype-specific interventions.

### 7.2. Digital Behavioral Monitoring and Phenotyping

Digital phenotyping is transforming apathy assessment by enabling continuous, real-world monitoring of motivation-related behaviors. Passive data from smartphones, wearables, and home-based sensors can quantify activity, social interaction, and engagement with minimal patient burden. Early studies show strong correlations between these digital markers and traditional apathy scales, supporting their use in real-time symptom tracking [62, 63].

Beyond assessment, platforms combining gamified cognitive exercises, VR environments, and AI-driven behavioral prompts may enhance engagement while generating actionable clinical data.

### 7.3. Pharmacological Innovation

Novel therapeutic strategies targeting apathy's neurochemical basis are emerging, including

• D3 receptor agonists (e.g., pramipexole): Improved receptor selectivity and favorable tolerability in motivational dysfunction [64].
• Triple reuptake inhibitors (TRIs): Simultaneously modulating dopamine, norepinephrine, and serotonin for treatment-resistant cases with apathy [65].
• Glutamatergic and orexinergic modulators: Experimental approaches to enhance reward sensitivity and arousal [66]. Future trials must designate apathy as a primary endpoint and stratify participants by dominant subtype for optimal efficacy evaluation.

### 7.4. Methodological Advancements

Priority areas include the following:

• Longitudinal studies mapping apathy progression across illness stages
• Multimodal neuroimaging integrated with behavioral and cognitive metrics
• Standardized, cross-culturally validated diagnostic tools
• Inclusion of caregiver perspectives and real-world functional outcomes in clinical trials

Such advances will support unified diagnostic frameworks, robust outcome measures, and personalized interventions across neuropsychiatric conditions.

## 8. Conclusion

Apathy is a multifaceted and clinically significant syndrome that transcends traditional diagnostic boundaries in neuropsychiatry. It contributes to functional decline, poor treatment adherence, and reduced quality of life—yet remains underrecognized and undertreated. Current evidence supports apathy as a distinct neurobehavioral entity with unique neural correlates and prognostic value. While nonpharmacological strategies currently provide the most consistent benefits, emerging pharmacological and digital interventions hold promise for more targeted, scalable solutions. Optimizing care will require a subtype-specific, multidimensional framework informed by validated assessment tools, biomarker insights, and patient-centered outcomes.

Future research must prioritize integrative, personalized, and technology-driven strategies to transform apathy from a secondary observation into a primary therapeutic target, bridging neurobiological insights with real-world functionality to improve outcomes in neuropsychiatric disorders.

## Figures and Tables

**Table 1:** Subtypes of apathy and their neurobiological and clinical characteristics.

**Subtype**	**Associated neuroanatomical regions**	**Key clinical features**
Behavioral apathy	Mesolimbic system (e.g., ventral tegmental area and nucleus accumbens)	Lack of initiative and spontaneity in daily activities
Cognitive apathy	Dorsolateral prefrontal cortex (DLPFC), caudate nucleus	Impaired planning, decision-making, distractibility
Emotional apathy	Orbitofrontal cortex (OFC), anterior cingulate cortex (ACC), insula, ventral striatum (VS)	Reduced empathy, diminished emotional responsiveness

**Table 2:** Major brain regions implicated in apathy: Functional roles and clinical associations.

**Brain region**	**Primary function**	**Association with apathy**
Dorsolateral prefrontal cortex (DLPFC)	Executive planning, cognitive flexibility	Impaired executive functioning; reduced goal-setting
Anterior cingulate cortex (ACC)	Effort-based decision-making, action initiation	Decreased motivation; deficits in reward processing
Orbitofrontal cortex (OFC)	Social cognition, reward valuation	Emotional blunting; diminished social engagement
Ventral striatum	Reward anticipation, hedonic processing	Lack of anticipatory pleasure; reduced drive for action
Medial prefrontal cortex (mPFC)	Self-referential processing, initiation of actions	Difficulty initiating voluntary behavior; amotivation

**Table 3:** Distinguishing features of apathy and commonly misidentified clinical conditions.

**Condition**	**Descriptive features**	**Key distinguishing characteristics**
Apathy	Diminished motivation, emotional blunting, reduced initiation of behavior	Absence of sadness, guilt, or hopelessness; the individual is “not sad, just indifferent”
Depression	Depressed mood, anhedonia, feelings of guilt, suicidal ideation	Prominent emotional distress and inner suffering; negative affect is central
Fatigue	Physical and mental exhaustion, reduced energy levels	Motivation is intact, but effort is limited due to perceived or actual lack of energy
Dementia	Memory impairment, disorientation, language and executive dysfunction	Apathy may coexist with dementia but is a distinct syndrome when present independently

**Table 4:** Commonly used scales for the assessment of apathy.

**Scale name**	**Clinical application**	**Subtype differentiation**	**Clinical advantages**
Apathy Evaluation Scale (AES)	Neurological and psychiatric disorders	Yes	Widely used; demonstrates good reliability and validity across settings
Lille Apathy Rating Scale (LARS)	Parkinson's disease, frontotemporal dementia (FTD)	Yes	Strong psychometric properties; effective in distinguishing apathy subtypes
Neuropsychiatric Inventory–Apathy Subscale (NPI-Apathy)	Dementia, particularly Alzheimer's disease	No	Brief administration time; integrated into broader behavioral assessment
Dimensional Apathy Scale (DAS)	General clinical and research settings	Yes	Reflects the multidimensional structure of apathy with domain-specific subscales

**Table 5:** Apathy prevalence and key clinical features across neuropsychiatric disorders.

**Disorder**	**Prevalence of apathy**	**Clinical prominence**	**Distinctive characteristics**
Frontotemporal dementia (bvFTD)	70%–100%	Core early symptom	Behavior driven by external cues; marked social disinterest
Schizophrenia	~51%	Core negative symptom	Motivational loss with emotional neutrality; lacks depressive affect
Major depressive disorder	40%–60%	May co-occur or present as distinct	“Not sad, just indifferent”; reduced initiative without sadness or guilt
Multiple sclerosis	20%–50%	Often underrecognized	Frequently associated with executive dysfunction and cognitive inertia
Parkinson's disease	20%–60%	Common nonmotor symptom	Linked to dopamine depletion; may respond poorly to standard dopaminergic therapy
Huntington's disease	50%–70%	Present even in prodromal stage	Strongly linked to striatal degeneration; rarely self-reported due to poor insight
Stroke	20%–60%	Hinders rehabilitation	Clinical features vary with lesion location; often persists into chronic phase

**Table 6:** Brain regions implicated in apathy across neuropsychiatric disorders.

**Disorder**	**Key brain regions**	**Clinical and neurobiological notes**
Frontotemporal disorders	ACC, OFC, mPFC, DLPFC	Impaired effort–reward evaluation; external cue dependency common
Schizophrenia	DLPFC, ACC, basal ganglia	White matter disruption; cognitive apathy predominant
Major depressive disorder	Ventral striatum, mPFC, caudal ACC	Dissociation of apathy from emotional depression; impaired reward anticipation
Multiple sclerosis	DLPFC, basal ganglia, ACC, insula	Strong link with executive dysfunction and motivational inertia
Huntington's disease	Striatum (caudate and putamen), prefrontal cortex, thalamus	Apathy often predates motor symptoms; reflects frontostriatal and thalamocortical dysfunction
Stroke	Dorsal ACC, DLPFC, globus pallidus, medial frontal cortex	Lesion-specific manifestation; apathy impairs neuroplasticity and recovery
Alzheimer's disease	ACC, OFC, ventral striatum	Correlates with tau and amyloid pathology; partial response to cholinergic therapy

**Table 7:** Pharmacological interventions in apathy.

**Drug class/agent**	**Target condition(s)**	**Clinical notes**
Dopamine agonists (e.g., *ropinirole* and *pramipexole*)	Parkinson's disease	Among the most extensively studied; act on D2/D3 receptors; shown to reduce motivational indifference
Cholinesterase inhibitors (*donepezil* and *rivastigmine*)	Alzheimer's, Parkinson's disease	Modest but statistically significant benefits for apathy; impact on global function remains limited
Methylphenidate	Alzheimer's disease	Promising for apathy symptom reduction; limited improvements in ADLs and caregiver burden
Modafinil	MS, Alzheimer's disease	Shows partial benefits; mechanism via dopaminergic activation; long-term efficacy unproven
Bupropion	Depression, MS, AD	Despite dopaminergic activity, clinical results are inconsistent and often subtherapeutic for apathy

**Table 8:** Structured clinical practice guideline for apathy management.

**Stage**	**Recommended clinical practice**
Screening	Use validated assessment tools such as the Apathy Evaluation Scale (AES), Neuropsychiatric Inventory (NPI), or a structured clinical interview to detect apathy
Subtype identification	Identify the dominant apathy subtype: *Cognitive*, *emotional*, or *behavioral*, to guide individualized intervention planning
Comorbidity assessment	Systematically evaluate for depression, fatigue, and cognitive impairment to improve diagnostic accuracy and avoid symptom overlap
Treatment planning	First-line: Initiate nonpharmacological interventions such as behavioral activation, structured exercise, and environmental enrichment. Second-line: Introduce pharmacological treatments (e.g., dopaminergic agents and bupropion) based on the underlying condition and apathy subtype
Monitoring and feedback	Routinely monitor goal-directed behavior, functional performance, and treatment response using structured tools; adjust interventions accordingly

## Data Availability

Data sharing is not applicable to this article as no new data were created or analyzed in this study.
